# Análise dos fatores de risco relacionados às amputações maiores e menores de membros inferiores em hospital terciário

**DOI:** 10.1590/1677-5449.008916

**Published:** 2017

**Authors:** Seleno Glauber de Jesus-Silva, João Pedro de Oliveira, Matheus Henrique Colepicolo Brianezi, Melissa Andreia de Moraes Silva, Arturo Eduardo Krupa, Rodolfo Souza Cardoso

**Affiliations:** 1 Faculdade de Medicina de Itajubá – FMIt, Itajubá, MG, Brasil.

**Keywords:** amputação, fatores de risco, isquemia, gangrena, diabetes mellitus, estudos transversais

## Abstract

**Contexto:**

As amputações dos membros inferiores, sejam definidas como maiores ou menores, são um grave problema de saúde, com altos índices de morbimortalidade e de relevante impacto social. Diferentes características clínicas dos pacientes parecem estar relacionadas aos diferentes tipos de amputação realizados.

**Objetivos:**

Analisar os fatores de risco presentes em pacientes submetidos a amputações de membros inferiores em hospital terciário.

**Métodos:**

Estudo retrospectivo, transversal, envolvendo 109 pacientes submetidos a amputação de membro inferior em um período de 31 meses, através da análise de gênero e idade, 15 dados clínicos e cinco parâmetros laboratoriais presentes no momento da admissão. Os dados foram submetidos a estatística descritiva e comparativa através do teste *t* de Student não pareado (para variáveis numéricas), e dos testes de Mann-Whitney e exato de Fisher (para variáveis categóricas).

**Resultados:**

Das 109 amputações realizadas, 59 foram maiores e 50 menores. A maioria dos pacientes era do gênero masculino (65%), e a média de idade foi de 65 anos (mín. 39, máx. 93). Dentre os fatores de risco observados, idade avançada, acidente vascular encefálico, isquemia, sepse e níveis baixos de hemoglobina e hematócrito estavam estatisticamente mais relacionados às amputações maiores (p < 0,05). Diabetes melito, neuropatia e pulsos distais palpáveis foram fatores mais associados às amputações menores.

**Conclusões:**

Os níveis das amputações de membros inferiores estão relacionados a diferentes fatores de risco. Os quadros isquêmicos mais graves e de maior morbidade estiveram associados a amputações maiores, enquanto a neuropatia e perfusão preservada, mais relacionados às amputações menores.

## INTRODUÇÃO

A amputação de um membro é um dos recursos terapêuticos mais antigos da medicina e significa a retirada total ou parcial, geralmente cirúrgica, de uma extremidade. Estima-se na literatura que sua incidência mundial varie de 2,8 a 43,9/10^5^ habitantes/ano^1^, enquanto no Brasil foi observada uma incidência de 13,9/10^5^ habitantes/ano. As amputações de membros inferiores correspondem a 85% do total e causam um grande impacto socioeconômico, com perda da capacidade laboral, da socialização e da qualidade de vida, além de complicações como hematoma, infecções, necrose, contraturas, neuromas, dor fantasma e reinternações, demonstrando ser um importante problema de saúde pública^2^^-^ 4.

No total, 80% de todas amputações de membros inferiores ocorrem em indivíduos adultos. Os principais fatores de risco relacionados são diabetes melito, hipertensão arterial, tabagismo, dislipidemia, idade avançada, insuficiência renal crônica, estados de hipercoagubilidade e fatores genéticos^5^^,^ 6. Metade dos casos de amputação ocorre em diabéticos^7^, seguidos de pacientes com aterosclerose não diabética, embolias e tromboses arteriais maciças. Pacientes diabéticos com neuropatia e/ou isquemia são mais suscetíveis à ulceração e à infecção, o que geralmente resulta em amputação^8^. Os traumatismos e os tumores malignos são responsáveis por, respectivamente, 10,6% e 5,8% das amputações ocorridas em membros inferiores^2^. Apesar do aumento do número de intervenções de revascularização, alguns trabalhos indicam que a prevalência de amputações se manteve inalterada devido ao aumento dos casos de aterosclerose e diabetes melito e ao envelhecimento da população^9^.

As amputações maiores são geralmente definidas como aquelas realizadas acima do nível do tornozelo, sejam transtibiais, transfemorais, desarticulações de joelho ou desarticulações de quadril, enquanto as menores são aquelas restritas aos pododáctilos ou ao nível do pé (sejam amputações transmetatársicas, desarticulações tarsometatársicas ou de Lisfranc, ou desarticulações médio-társicas ou de Chopart)^10^. As amputações menores são geralmente melhor aceitas por permitir a deambulação sem necessariamente o uso de prótese^11^. As taxas de mortalidade são diferentes para ambos os grupos, variando de 22% em 1 ano para as amputações menores^12^ até 21% em 1 mês a 52% em 1 ano para as maiores^13^.

O presente estudo tem como objetivo determinar os principais fatores de risco associados aos diferentes tipos de amputações de membros inferiores (maiores e menores) em pacientes tratados por equipe de cirurgia vascular e endovascular em hospital terciário.

## MATERIAIS E MÉTODOS

Trata-se de um estudo retrospectivo, realizado através da análise de prontuários de 109 pacientes submetidos a amputações maiores e menores de membros inferiores no período de julho de 2013 a janeiro de 2016 em serviço de cirurgia vascular e endovascular em hospital terciário. O estudo foi aprovado pelo Comitê de Ética em Pesquisa da instituição sob o número 1.290.602. A amostra foi calculada com nível de significância α = 5% e poder do teste β = 80%. Foram pesquisados e inseridos em um banco de dados criado em planilha eletrônica 15 dados clínicos, além de idade e gênero, e cinco parâmetros laboratoriais.

A hipertensão arterial sistêmica foi definida como pressões maiores que 140 × 90 mmHg ou uso contínuo de anti-hipertensivos, diabetes melito como glicemia de jejum > 106 mg/dL ou uso de hipoglicemiantes, insuficiência renal como *clearance* de creatinina < 60 mL/min ou creatinina sérica > 1,6 mg/dL, doença arterial obstrutiva periférica como índice tornozelo-braquial < 0,9 ou sinais clínicos evidentes de oclusão arterial, e a neuropatia como presença de mal perfurante plantar, deformidade óssea ou osteoartropatia de Charcot. Outros parâmetros pesquisados foram tabagismo; história de infarto agudo do miocárdio ou de acidente vascular encefálico (AVE); revascularização prévia do membro (relacionada ou não à doença atual); amputação prévia (qualquer amputação em nível mais distal na mesma extremidade); arritmia emboligênica – mais precisamente fibrilação atrial; infecção evidente no membro (presença de abscesso, necrose, supuração ou mal perfurante plantar com sequestro); sepse; presença de pulso pedioso e/ou tibial posterior; e pressão arterial sistólica e diastólica. A sepse foi definida como a presença de pelo menos dois dos seguintes fatores associados a uma infeção evidente: febre > 38 °C, frequência cardíaca > 90 batimentos por minuto, taquipneia > 20 ipm e leucocitose > 12.000/mm^3^). Eritrograma e leucograma à admissão foram anotados, assim como os níveis de glicemia e a creatinina. Foram excluídos previamente do estudo 15 pacientes com prontuários incompletos e foi considerada somente a primeira internação de cada paciente.

A estatística descritiva entre os grupos foi seguida do teste *t* de Student não pareado bicaudal (para variáveis numéricas) e do teste de Mann-Whitney e exato de Fisher (para variáveis categóricas). A associação entre os fatores de risco e o tipo de amputação foi realizada através do teste de correlação linear de Pearson. O software estatístico utilizado foi o Bioestat versão 5.3, utilizando IC de 95% e significância estatística para p < 0,05.

## RESULTADOS

Do total de 109 procedimentos de amputações realizados, 59 foram maiores e 50 menores. Das amputações maiores, 39 foram transfemorais (21 à esquerda e 18 à direita) e 20 transtibiais (11 à esquerda e nove à direita). Em relação às amputações menores, duas foram transtársicas, oito transmetatársicas e 40 de pododáctilos.

A maioria dos pacientes era do gênero masculino (n = 71; 65%), e a média de idade foi de 65 anos (mín. = 39, máx. = 93). Do total de amputações maiores, seis foram decorrentes de oclusão arterial aguda (10,1%), e os demais 53 casos (89,8%) foram decorrentes de doença arterial oclusiva periférica e/ou complicações do pé diabético. Todos os pacientes submetidos a amputação menor apresentavam aterosclerose descompensada e/ou pé diabético infectado. Foram registrados em prontuário 10 óbitos de pacientes submetidos a amputações maiores, e nenhum nas amputações menores, num período de seis meses.

A Tabela 1 relaciona os fatores de risco estudados tanto na amostra total quanto nos subgrupos. Não houve diferença entre os grupos em relação à prevalência de revascularização prévia do membro (maiores 31% *versus* menores 22%, p = 0,83).

**Tabela 1:** Características epidemiológicas e fatores de risco presentes na amostra e em cada um dos subgrupos analisados.

**Fatores de risco**	**Total**		**Amputações maiores**		**Amputações menores**		**p**
	**n**	**%**	**n**	**%**	**n**	**%**	
**Gênero**							
**Masculino**	71	65	36	61	35	70	0,4203
**Feminino**	38	35	23	39	15	30	
**Idade (anos)**	65,3	12,1 (DP)	69	13,1 (DP)	62	9,7 (DP)	0,0023
**HAS**	86	79	50	85	36	72	0,1567
**DM**	82	75	38	64	44	88	0,0069
**Tab**	33	30	17	29	16	32	0,8347
**IAM**	14	12	7	12	7	14	0,7802
**IR**	13	12	9	15	4	8	0,3747
**Fibrilação atrial**	22	20	12	20	10	20	1,0000
**AVE**	19	18	16	27	3	6	0,0047
**Revasc. membro**	29	28	18	31	11	22	0,8371
**Amputação. prévia**	26	25	17	29	9	18	0,2597
**Isquemia**	69	63	46	78	23	46	0,0007
**Infecção**	79	72	40	68	39	78	0,2847
**Sepse**	16	15	14	24	2	4	0,0053
**Pulsos distais palpáveis**	40	37	10	17	30	60	0,0001
**Neuropatia**	34	32	13	22	21	43	0,0374
**PAS (mmHg)**	136	16,5 (DP)	136	17,7 (DP)	130	15,8 (DP)	0,5608
**PAD (mmHg)**	84	10,4 (DP)	83	9,3 (DP)	80	9,7 (DP)	0,4512
**Leucograma (leucócitos/mm^3^)**	12.693	4.920 (DP)	13.207	4.158 (DP)	11.200	5.585 (DP)	0,4379
**Glicose (mg/dL)**	169	96 (DP)	155	93 (DP)	197	99 (DP)	0,1806
**Creatinina (mg/dL)**	1,43	1,22 (DP)	1,04	1,29 (DP)	1,10	1,20 (DP)	0,5907
**Hemoglobina (g/dL)**	11,9	2,5 (DP)	11,6	2,9 (DP)	12,8	1,9 (DP)	0,0063
**Hematócrito (%)**	35,4	7,1 (DP)	34,6	8,2 (DP)	37,7	5,2 (DP)	0,0136

HAS: hipertensão arterial sistêmica; DM: diabetes melito; Tab: tabagismo; IAM: infarto agudo do miocárdio; IR: insuficiência renal; AVE: acidente vascular encefálico; Revasc. membro: revascularização de membro; PAS: pressão arterial sistólica; PAD: pressão arterial diastólica; DP: desvio-padrão.

**Figura 1:**
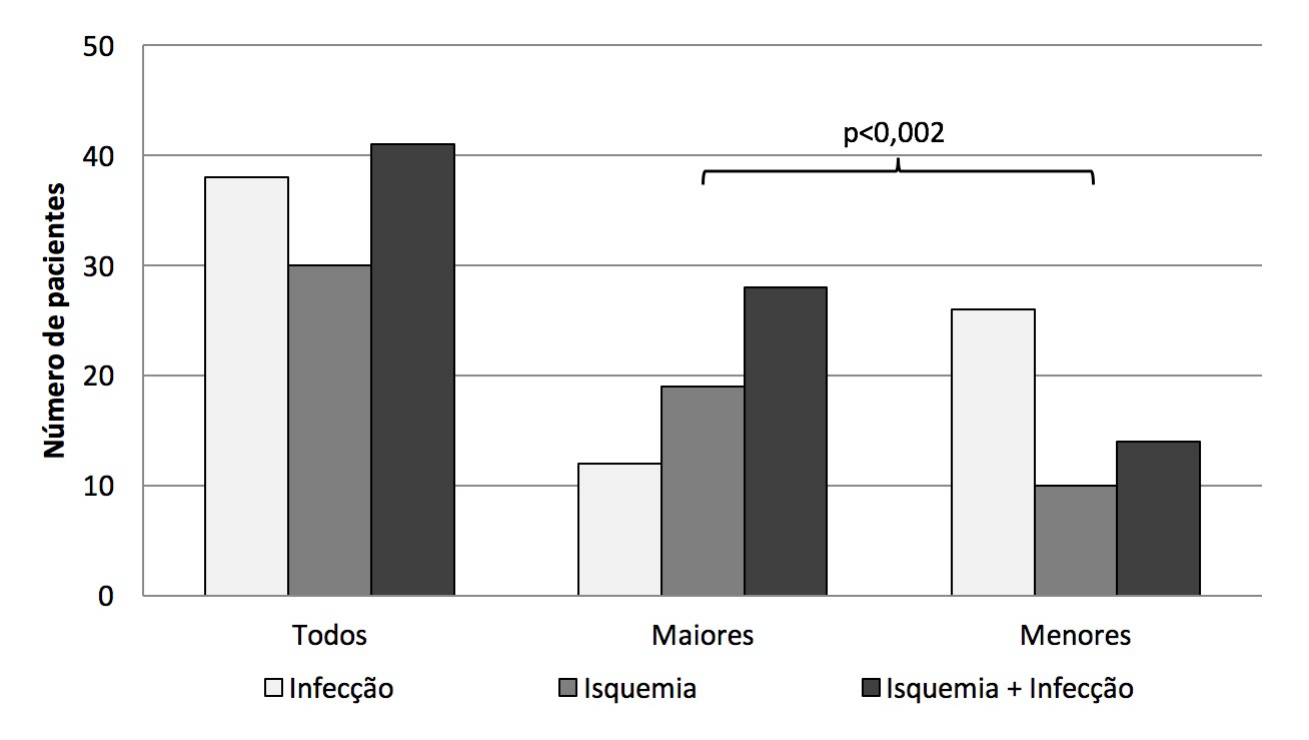
Gráfico comparativo entre os tipos de amputações realizadas e as etiologias primárias associadas. Houve maior prevalência de infecção no grupo de amputações menores e de isquemia nas amputações maiores (teste de Mann-Whitney).

A análise comparativa entre os grupos de amputações revelou uma tendência de as amputações maiores serem mais associadas à isquemia e menos à infecção, resultado contrário ao observado nas amputações menores (Figura 1).

Os testes de correlação linear de Pearson entre os fatores de risco estudados e o tipo de amputação realizada estão descritos na Tabela 2. Idade avançada, AVE, isquemia, sepse e amputação secundária foram mais relacionados às amputações maiores, enquanto o diabetes melito, neuropatias, presença de pulsos distais palpáveis e níveis maiores de hemoglobina e hematócrito estavam mais presentes nos pacientes submetidos a amputações menores.

**Tabela 2:** Correlação linear de Pearson para os fatores de risco analisados em relação ao tipo de cirurgia realizada.

**Fator de risco**	**r**	**IC (mín.-máx.)**		**p**
Pulsos distais palpáveis	–0,4451	–0,58	–0,28	< 0,0001
Hemoglobina	–0,2837	–0,45	–0,10	0,0028
Diabetes melito	–0,2723	–0,44	–0,09	0,0041
Hematócrito	–0,2585	–0,43	–0,07	0,0066
Neuropatia	–0,2344	–0,40	–0,05	0,0141
Idade	0,2893	0,11	0,45	0,0023
Acidente vascular encefálico	0,2774	0,09	0,44	0,0035
Sepse	0,2778	0,09	0,44	0,0034
Isquemia	0,3111	0,15	0,49	0,0004

IC: intervalo de confiança. Fatores de risco com valor de *r* positivos são mais relacionados às amputações maiores, enquanto valores de *r* negativos apontam maior relação às amputações menores.

## DISCUSSÃO

As amputações de membros, seja no nível dos pododáctilos, ou envolvendo a perda parcial ou total do membro, trazem consigo desafios clínicos e sociais para os pacientes a elas submetidos, mesmo que em diferentes níveis. Determinar as características clínicas e os fatores de risco de cada grupo é fundamental para compreender o processo que leva à perda do membro.

A incidência de amputação é variável no mundo. Metanálise envolvendo publicações de 1989 a 2010 detectou incidência de 5,8 a 31 casos/10^5^ habitantes na população geral e de 46,1 a 9.600 casos/10^5^ habitantes nos diabéticos^14^. No Brasil, estudo populacional envolvendo 5.539 indivíduos submetidos a amputações maiores observou uma incidência de 9,7 casos/10^5^ habitantes na faixa de 30 a 89 anos e de 29,5 casos/10^5^ habitantes de 55 a 74 anos, valores que aumentaram significativamente quando estudados somente os diabéticos (45,98 e 92,19 casos/10^5^ habitantes, respectivamente)^5^. A média de idade para os pacientes estudados foi semelhante à de outros estudos populacionais, de aproximadamente 65 anos^5^^,^ 15.

O mesmo estudo populacional brasileiro observou distribuição semelhante das etiologias, com diabetes e doença arterial crônica periférica acometendo a grande maioria dos casos (90,7%), seguido do trauma (5,6%), osteomielite (1,7%), gangrena gasosa (1,2%) e neoplasias (0,8%)^5^. Em comparação, outro estudo nacional com pacientes em programação de reabilitação observou maior número de causas traumáticas (33%) e menor de causas vasculares (51,5%)^2^. Esse viés foi provavelmente determinado pelo tipo de especialidade e de indivíduos envolvidos, uma vez que pacientes encaminhados para reabilitação geralmente possuem doença vascular menos grave e são mais jovens. Estima-se que a real incidência de amputações maiores de membros no Brasil seja mais relacionado às causas vasculares, devido à alta prevalência da doença aterosclerótica e diabetes, com consequente menor encaminhamento para reabilitação.

A relação entre as taxas de amputação maiores e menores foi de cerca de 2:1, o que vai de encontro a estudos populacionais, que observaram taxas inversas de 1:2^16^. Além disso, sabe-se que existe uma variação ampla nas taxas de amputação, a depender das características socioeconômicas e de acesso ao atendimento médico^17^. Estimamos que a realidade encontrada no presente estudo reflete a demora no acesso ao atendimento médico e o precário nível socioeconômico, fazendo com que muitos casos fossem admitidos com nítida impossibilidade de preservação do nível infrapatelar e sequer de tentativa de revascularização.

Em relação aos fatores de risco estudados, observou-se que idade avançada, AVE prévio, amputação prévia, sepse, isquemia e anemia (níveis diminuídos de hemoglobina e hematócrito) estiveram estatisticamente mais relacionados às amputações maiores, enquanto diabetes, presença de pulso distal e neuropatia instalada estiveram mais relacionados às amputações menores. Tais características ressaltam os aspectos distintos entre ambos os grupos.

Estudo israelense retrospectivo envolvendo 594 diabéticos (dos quais 53,2% haviam sido submetidos a amputações maiores) relatou anemia, leucocitose, hipoalbuminemia e *clearance* de creatinina diminuído como fatores significativamente relacionados às amputações maiores, enquanto níveis mais elevados da hemoglobina glicosilada eram os únicos fatores relacionados às amputações menores^18^. Série semelhante comparativa de 97 pacientes na Holanda observou maior relação das amputações maiores com AVE e revascularização prévia do membro^9^. Estudo intra-hospitalar da Coreia do Sul revelou que diálise, mal perfurante plantar com acometimento ósseo, distúrbios gastrintestinais, úlceras de calcâneo, anemia e glicemia anormal são mais relacionados às amputações^19^. Níveis baixos de HDL também já foram estudados e implicados como fatores prognósticos para amputações de qualquer nível em diabéticos portadores de úlceras plantares^20^. A calcificação parietal e incompressibilidade vascular distal nos diabéticos, por sua vez, são considerados fatores prognósticos para amputação tão importantes quanto níveis diminuídos do índice tornozelo-braquial^21^.

O presente estudo identificou que níveis diminuídos de hematócrito e hemoglobina estiveram significativamente mais presentes nos pacientes submetidos a amputações maiores se comparados àqueles submetidos às menores. Publicação semelhante, envolvendo somente portadores de doença arterial oclusiva periférica, observou um risco significativamente mais elevado de amputação maior (*odds ratio*, OR = 1,56) e de morte e amputação (OR = 1,58) em 1 ano nos portadores de anemia em relação àqueles que apresentavam níveis normais de hemoglobina^22^. A presença de anemia, por outro lado, associada a transfusão sanguínea em pacientes submetidos a amputação, também é considerada um fator prognóstico negativo para a ocorrência de complicações como pneumonia, tromboembolismo venoso e tempo de internação prolongado^23^.

As infeções locais nos diabéticos, com úlceras plantares, edema e hiperemia, são sabidamente fatores de alto risco para amputações^8^^,^ 24. O presente estudo observou, entretanto, que somente a sepse está relacionada às amputações maiores, independentemente da presença de infecção local. Ressalta-se a importância da decisão terapêutica imediata nesses casos iniciais, evitando a progressão para o choque séptico e o óbito.

A presença de úlcera crônica no pé diabético configura um fator de risco adicional para o desenvolvimento das fasceítes necrotizantes e a ocorrência de amputações de membro. Estima-se que a fasceíte necrotizante afete até 4,9% dos diabéticos, e que a probabilidade de amputação chegue a 72,4% caso esses pacientes sejam também portadores de úlcera plantar^25^. Neste mesmo estudo, detectou-se que a hipoalbuminemia foi um fator determinante para a amputação, sendo encontrados níveis menores de albumina em pacientes submetidos a amputação maior se comparados àqueles submetidos a amputação menor (2,3 g/dL *versus* 2,6 g/dL, p = 0,002), e que altos graus da Classificação de Wagner (estágios 4 e 5) são mais relacionados à ocorrência de perda do membro.

A revascularização do membro ocorreu de forma semelhante em ambos os grupos (28% do total), porém não foi observada uma menor prevalência de amputações nos pacientes revascularizados. O benefício da revascularização na incidência de amputações, entretanto, já foi alvo de estudos de longo prazo^26^^-^ 28. Observamos, no entanto, através da regressão linear, que o principal fator determinante para a evolução entre amputação maior ou menor era a condição inicial de vascularização do membro. Estudos de caso-controle seriam melhores empregados para determinar com mais precisão a evolução natural dos pacientes revascularizados e não revascularizados, assim como a relação entre a amputação e as taxas de perviedade.

A diminuição global na incidência de amputações maiores foi observada em vários estudos, e está relacionada ao melhor controle do diabetes (apesar do aumento em sua incidência) e melhores estratégias governamentais de saúde pública, como a instalação de equipes multiprofissionais para o tratamento do pé diabético e expansão da rede de saúde^10^^,^ 14 ^,^ 15 ^,^ 29 ^-^ 31. Não foi possível determinar neste trabalho transversal e unicêntrico a eficácia das ações de saúde pública na ocorrência das amputações.

Dentre as limitações do presente estudo retrospectivo está a ausência de mais variáveis clínicas a serem estudadas, como obesidade, níveis de HDL, LDL, hemoglobina glicosilada e medicações em uso, em parte porque a coleta de dados dependeu do adequado preenchimento dos prontuários hospitalares. As taxas de mortalidade a curto e a médio prazo não foram consideradas precisas, o que poderia trazer dados relevantes acerca do impacto de cada tipo de amputação na sobrevida. A mortalidade quase nunca era um dado disponível em prontuário, exceto nos casos de óbito intra-hospitalar e naqueles que retornavam regularmente ao ambulatório para acompanhamento, uma vez que muitos dos pacientes analisados eram posteriormente tratados em suas cidades de origem, e os serviços de notificação não eram integrados. A reabilitação (protetização) também era um dado pouco acessível, pois era realizada em serviços de referência em outra cidade. A prevalência das principais comorbidades como diabetes, hipertensão e doença renal no presente trabalho foi maior em comparação a outras publicações semelhantes^25^^,^ 32 ^-^ 34, o que não só caracteriza a população estudada como de alto risco, mas também ressalta a carência de serviços públicos de prevenção de saúde na área geográfica estudada.

Em conclusão, observou-se que as amputações maiores e menores dos membros inferiores apresentam diferentes fatores de risco para o grupo populacional estudado. Pacientes submetidos a amputações transtibiais ou transfemorais são geralmente portadores de idade avançada, isquemia mais grave, anemia, sepse e/ou AVE, enquanto aqueles submetidos a amputações de pododáctilos ou ao nível do pé apresentam perfusão preservada e sinais claros de neuropatia diabética. Estudos multicêntricos envolvendo diversas especialidades cirúrgicas relacionadas às amputações (cirurgia vascular, ortopedia) e com o objetivo de determinar a mortalidade a curto e a médio prazo são fundamentais para a expansão do conhecimento acerca do real impacto das amputações nos pacientes em nosso meio.
