# Supplementary material for: Análise dos fatores de risco relacionados às amputações maiores e menores de membros inferiores em hospital terciário
Source: J Vasc Bras. 2017 Jan-Mar;16(1):16–22. [Article in Portuguese] doi: 10.1590/1677-5449.008916 (PMC5829687; doi:10.1590/1677-5449.008916)
Supplement: Translation

# Translation: Analysis of risk factors related to minor and major lower limb amputations at a tertiary hospital

**Authors:** Seleno Glauber de Jesus-Silva, João Pedro de Oliveira, Matheus Henrique Colepicolo Brianezi, Melissa Andreia de Moraes Silva, Arturo Eduardo Krupa, Rodolfo Souza Cardoso

**Affiliations:** 1 Faculdade de Medicina de Itajubá – FMIt, Itajubá, MG, Brazil.

**Keywords:** amputation, risk factors, ischemia, gangrene, diabetes mellitus, cross-sectional studies

## Abstract

**Background:**

Whether they are defined as minor or major, lower limb amputations constitute a severe health problem, causing high rates of morbidity and mortality and considerable social impact. Different patient clinical characteristics appear to be related to different types of amputations.

**Objectives:**

To analyze risk factors present in patients who underwent lower limb amputations at a tertiary hospital.

**Methods:**

This was a retrospective, cross-sectional study of 109 patients who underwent lower limb amputations over a period of 31 months, analyzing gender, age, 15 clinical data and five laboratory parameters present at the time of admission. Data were treated with descriptive statistics and compared using Student’s *t* test for unpaired samples (for numerical variables), the Mann-Whitney test, or Fisher’s exact tests (for categorical variables).

**Results:**

There were 59 major and 50 minor amputations out of a total of 109 performed. The majority of patients were male (65%) and mean age was 65 years (range 39 to 93). Risk factors that were statistically (p < 0.05) more related to major amputations were advanced age, stroke, ischemia, sepsis, and low hemoglobin and hematocrit levels. Diabetes mellitus, neuropathy, and palpable distal pulses were factors more strongly associated with minor amputations.

**Conclusions:**

Lower limb amputations at different levels are related to different risk factors. Ischemia of greater severity and morbidity was associated with major amputations, while neuropathy and preserved perfusion were more often related to minor amputations.

## INTRODUCTION

Limb amputation is one of the oldest treatment options available in medicine and consists of the total or partial removal, generally surgical, of an extremity. Estimates of incidence in the worldwide literature vary from 2.8 to 43.9/10^5^ inhabitants/year,^1^ while in Brazil an incidence of 13.9/10^5^ inhabitants/year has been observed. Lower limb amputations account for 85% of this total and have a major socioeconomic impact, with loss of the capacity to work, of socialization, and of quality of life, in addition to complications such as hematoma, infections, necrosis, contractures, neuromas, phantom pain, and re-admissions, making them a considerable public health problem.^2^^-^ 4

Overall, 80% of all lower limb amputations are performed on adults. The main associated risk factors are diabetes mellitus, arterial hypertension, smoking, dyslipidemia, advanced age, chronic renal failure, hypercoagulability, and genetic factors.^5^^,^ 6 Half of all amputations are performed on diabetics,^7^ followed by patients with non-diabetic atherosclerosis, massive arterial thrombosis and embolism. Diabetic patients with neuropathy and/or ischemia are more susceptible to ulceration and infection, which generally results in amputation.^8^ Traumatisms and malignant tumors are responsible for, respectively, 10.6% and 5.8% of lower limb amputations.^2^ Despite the increase in the number of revascularization interventions, some studies indicate that the prevalence of amputations has remained unaltered because of the increase in cases of atherosclerosis and diabetes mellitus and aging of the population.^9^

Major amputations are generally defined as those performed above the level of the ankle, whether transtibial, transfemoral, knee disarticulations or hip disarticulations, while minor amputations are those restricted to the toes or at the level of the foot (whether transmetatarsal amputations, tarsometatarsal or Lisfranc disarticulations, or mid-tarsal or Chopart disarticulations).^10^ Minor amputations are generally better accepted because they do not necessarily impose a need for prostheses to enable walking.^11^ Mortality rates differ between the two groups, varying from 22% within 1 year for minor amputations^12^ to 21% within 1 month up to 52% within 1 year for major amputations.^13^

The objective of this study is to determine the main risk factors associated with the different types of lower limb amputations (major and minor) in patients treated by a vascular and endovascular surgery team at a tertiary hospital.

## MATERIALS AND METHODS

This was a retrospective study analyzing the medical records for 109 patients who underwent major or minor lower limb amputations between July 2013 and January 2016 at a vascular and endovascular surgery service in a tertiary hospital. The study was approved by the institution’s Research Ethics Committee under number 1.290.602. The sample size was calculated to a significance level of α = 5% for a test power of β = 80%. Age, gender, 15 clinical data and five laboratory parameters were extracted from medical records and inserted in an electronic spreadsheet.

Systemic arterial hypertension was defined as pressures greater than 140 × 90 mmHg or continuous use of antihypertensive drugs, diabetes mellitus as fasting glycemia > 106 mg/dL or use of hypoglycemiants, renal failure as creatinine clearance < 60mL/min or serum creatinine > 1.6 mg/dL, peripheral arterial occlusive disease as an ankle-brachial index < 0.9 or evident clinical signs of arterial occlusion, and neuropathy as presence of plantar trophic ulcers, bone deformity, or Charcot osteoarthropathy. Other parameters recorded were smoking; history of acute myocardial infarction or stroke; prior revascularization of the limb (irrespective of whether or not it was related to the current condition); prior amputation (any amputation at a level distal to that of the current extremity); emboligenic arrhythmia – more specifically atrial fibrillation; evident infection of the limb (presence of abscess, necrosis, suppuration, or plantar trophic ulcers with sequestration); sepsis; presence of pedal pulse and/or posterior tibial pulse; and systolic and diastolic arterial blood pressures. Sepsis was defined as presence of at least two of the following factors associated with evident infection: fever > 38 °C, heart rate > 90 beats per minute, tachypnea >20 breaths per minute and leukocytosis >12,000/mm^3^). Red and white blood cell counts on admission were recorded, as were glycemia and creatinine levels. Before analysis, 15 patients with incomplete medical records were excluded from the study and only the first admission for each patient was included.

Descriptive statistics were calculated for the subsets and then the two-tailed Student’s *t* test for unpaired samples (for numerical variables), the Mann-Whitney test or Fisher’s exact test (for categorical variables) were applied. Associations between risk factors and type of amputation were detected using Pearson’s linear correlation test. The statistical software employed was Bioestat version 5.3, with a 95% confidence interval and statistical significance cutoff of p < 0.05.

## RESULTS

There were 59 major and 50 minor amputations out of a total of 109 performed. Among the major amputations, 39 were transfemoral (21 on the left and 18 on the right) and 20 were transtibial (11 on the left and nine on the right). The minor amputations were two transtarsal, eight transmetatarsal, and 40 toe amputations.

The majority of patients were male (n = 71; 65%) and the mean age of the sample was 65 years (range = 39 to 93). Six of the 59 major amputations were caused by acute arterial occlusion (10.1%) and the remaining 53 cases (89.8%) were the result of occlusive peripheral arterial disease and/or complications of the diabetic foot. All the patients who had minor amputations had decompensated atherosclerosis and/or an infected diabetic foot. There were 10 recorded deaths of patients who had had major amputations and no death of patients who underwent minor amputations, within a six-month period.

Table 1 lists the statistics for the risk factors studied, in the entire sample and in the subsets. The prevalence of prior revascularization of the limb did not differ between groups (major amputations 31% vs. minor amputations 22%, p = 0.83).

**Table 1:** Epidemiological characteristics and risk factors in the whole sample and in each of the subsets analyzed.

**Risk factors**	**Total**		**Major amputations**		**Minor amputations**		**p**
	**n**	**%**	**n**	**%**	**n**	**%**	
Gender							
Male	71	65	36	61	35	70	0.4203
Female	38	35	23	39	15	30	
Age (years)	65.3	12.1 (SD)	69	13.1 (SD)	62	9.7 (SD)	0.0023
SAH	86	79	50	85	36	72	0.1567
DM	82	75	38	64	44	88	0.0069
Smoking	33	30	17	29	16	32	0.8347
AMI	14	12	7	12	7	14	0.7802
KF	13	12	9	15	4	8	0.3747
Atrial fibrillation	22	20	12	20	10	20	1.0000
stroke	19	18	16	27	3	6	0.0047
Limb revasc.	29	28	18	31	11	22	0.8371
Previous amputation	26	25	17	29	9	18	0.2597
Ischemia	69	63	46	78	23	46	0.0007
Infection	79	72	40	68	39	78	0.2847
Sepsis	16	15	14	24	2	4	0.0053
Palpable distal pulses	40	37	10	17	30	60	0.0001
Neuropathy	34	32	13	22	21	43	0.0374
SBP (mmHg)	136	16.5 (SD)	136	17.7 (SD)	130	15.8 (SD)	0.5608
DBP (mmHg)	84	10.4 (SD)	83	9.3 (SD)	80	9.7 (SD)	0.4512
WBC (leukocytes/mm^3^)	12,693	4,920 (SD)	13,207	4,158 (SD)	11,200	5,585 (SD)	0.4379
Glucose (mg/dL)	169	96 (SD)	155	93 (SD)	197	99 (SD)	0.1806
Creatinine, (mg/dL)	1.43	1.22 (SD)	1.04	1.29 (SD)	1.10	1.20 (SD)	0.5907
Hemoglobin, (g/dL)	11.9	2.5 (SD)	11.6	2.9 (SD)	12.8	1.9 (SD)	0.0063
Hematocrit (%)	35.4	7.1 (SD)	34.6	8.2 (SD)	37.7	5.2 (SD)	0.0136

SAH: systemic arterial hypertension; DM: diabetes mellitus; AMI: acute myocardial infarction; KF: kidney failure; Limb revasc.: limb revascularization; SBP: systolic arterial blood pressure; DBP: diastolic arterial blood pressure; WBC: white blood cell count; SD: standard deviation.

Comparative analysis of the two types of amputations revealed a tendency for major amputations to be more strongly associated with ischemia and less so with infection, while the opposite was observed for minor amputations (Figure 1).

**Figure 1:** Comparative graph illustrating types of amputations and primary etiologies. There was a higher prevalence of infection in the minor amputation subset and a higher prevalence of ischemia in the major amputation subset (Mann-Whitney test).

The results for Pearson’s linear correlation test between risk factors and type of amputation are shown in Table 2. Advanced age, stroke, ischemia, sepsis, and secondary amputation were more strongly related to major amputations, while diabetes mellitus, neuropathies, palpable distal pulses, and higher levels of hemoglobin and hematocrit were more likely to be present in patients who underwent minor amputations.

**Table 2:** Pearson linear correlations between risk factors analyzed and type of surgery performed.

**Risk factor**	**r**	**CI (min.-max.)**		**p**
Palpable distal pulses	–0.4451	–0.58	–0.28	< 0.0001
Hemoglobin	–0.2837	–0.45	–0.10	0.0028
Diabetes mellitus	–0.2723	–0.44	–0.09	0.0041
Hematocrit	–0.2585	–0.43	–0.07	0.0066
Neuropathy	–0.2344	–0.40	–0.05	0.0141
Age	0.2893	0.11	0.45	0.0023
Stroke	0.2774	0.09	0.44	0.0035
Sepsis	0.2778	0.09	0.44	0.0034
Ischemia	0.3111	0.15	0.49	0.0004

CI: confidence interval. Risk factors with positive *r* values have a stronger relationship with major amputations, while negative *r* values indicate a stronger relationship with minor amputations.

## DISCUSSION

Limb amputations, whether at the level of the toes, or involving partial or total loss of the limb, impose clinical and social challenges on the patients subjected to them, although to different degrees. Determination of the clinical characteristics and risk factors of each group is fundamental to understanding the processes that culminate in limb loss.

Worldwide, incidence rates of amputation are variable. A meta-analysis of publications from 1989 to 2010 found incidence rates of 5.8 to 31 cases/10^5^ inhabitants in the general population and of 46.1 to 9,600 cases/10^5^ inhabitants among diabetics.^14^ A population-based study in Brazil investigating 5,539 people who underwent major amputations observed an incidence of 9.7 cases/10^5^ inhabitants in an age range of 30 to 89 years and of 29.5 cases/10^5^ inhabitants aged 55 to 74 years and the figures were even higher for diabetics (45.98 and 92.19 cases/10^5^ inhabitants, respectively).^5^ The mean age of patients studied was similar to that for other population-based studies, at approximately 65 years.^5^^,^ 15

The same Brazilian population study observed a similar distribution of etiologies, with diabetes and chronic peripheral arterial disease present in the great majority of cases (90.7%), followed by trauma (5.6%), osteomyelitis (1.7%), gas gangrene (1.2%), and neoplasms (0.8%).^5^ In comparison, a different Brazilian study investigating patients on a rehabilitation program observed higher numbers of traumatic causes (33%) and lower rates of vascular causes (51.5%).^2^ This difference was probably because of the type of specialty and the patients involved, since patients referred for rehabilitation generally have less severe vascular disease and are younger. It is estimated that the true incidence of major limb amputations in Brazil is more related to vascular causes, due to the high prevalence of atherosclerotic disease and diabetes, with consequent lower rates of referral for rehabilitation.

The ratio of major to minor amputations was around 2:1, which is in contrast with the results of population studies, which have observed the inverse ratio of 1:2.^16^ Additionally, it is known that there is a wide range of variation in amputation rates, depending on socioeconomic characteristics and access to medical care.^17^ We hypothesize that the scenario observed in the present study reflects delays in access to medical care and low socioeconomic status, which mean that many cases are admitted at a point at which preservation at the infrapatellar level is evidently impossible and revascularization attempts are equally futile.

With relation to the risk factors studied, it was observed that advanced age, previous stroke, prior amputation, sepsis, ischemia and anemia (low hemoglobin and hematocrit levels) were statistically more related to major amputations while diabetes, palpable distal pulses, and preexisting neuropathy were more related to minor amputations. These characteristics highlight the two groups’ distinct characteristics.

A retrospective study in Israel that analyzed 594 diabetics (53.2% of whom had undergone major amputations) reported anemia, leukocytosis, hypoalbuminemia, and reduced creatinine clearance as factors significantly related to major amputations, while higher glycosylated hemoglobin levels was the only factor related to minor amputations.^18^ A similar comparative study of a 97-patient series in Holland observed stronger relationships between major amputations and stroke and prior revascularization of the limb.^9^ An intrahospital study in South Korea revealed that dialysis, plantar ulcers with bone involvement, gastrointestinal disorders, ulcers of the calcaneus, anemia, and abnormal glycemia were most related to amputations.^19^ Low HDL levels have also been studied and implicated as prognostic factors for amputations at all levels in diabetics with plantar ulcers.^20^ In turn, arterial calcification and distal vascular incompressibility in diabetics are considered prognostic factors of amputation equally as important as the ankle-brachial index.^21^

The present study identified that reduced hematocrit and hemoglobin levels were significantly more likely to be present in patients who underwent major amputations compared to those subjected to minor amputations. A similar publication, involving only patients with occlusive peripheral arterial disease, observed a significantly higher risk of major amputation (odds ratio [OR] = 1.56) and of death and amputation (OR = 1.58) at 1 year among those with anemia, in relation to those who had normal hemoglobin levels.^22^ On the other hand, the presence of anemia associated with blood transfusions in patients who undergo amputation is also considered a negative prognostic factor for occurrence of complications such as pneumonia, venous thromboembolism, and prolonged hospital stay.^23^

In diabetics, local infections, with plantar ulcers, edema, and hyperemia, are known high-risk factors for amputations.^8^^,^ 24 However, the present study observed that only sepsis was related to major amputations, irrespective of the presence of local infection. In these cases, the importance of taking a treatment decision immediately to prevent progression to septic shock and death cannot be overstated.

Chronic ulcers on a diabetic foot are an additional risk factor for development of necrotizing fasciitis and progression to amputation of the limb. It is estimated that necrotizing fasciitis affects up to 4.9% of diabetics, and that the probability of amputation reaches 72.4% in patients who also have plantar ulcers.^25^ The same study detected that hypoalbuminemia was a determinant factor for amputation, observing that albumin levels were lower in patients who underwent major amputations than in patients who had minor amputations (2.3 g/dL vs. 2.6 g/dL, p = 0.002) and that high grades on the Wagner Classification (stages 4 and 5) had a stronger association with loss of the limb.

Rates of revascularization of the limb did not differ between the two groups (28% of the entire sample) and the prevalence of amputations was not lower among revascularized patients. However, the benefits of revascularization in terms of incidence of amputations has already been investigated in long-term studies.^26^^-^ 28 Notwithstanding, we conducted a linear regression analysis that showed that the principal factor that determined progression to major or minor amputation was the initial vascularization status of the limb. Case-control studies would be better for determination with greater precision of the natural progression of revascularized and non-revascularized patients and the relationship between amputation and patency rates.

Several different studies have observed reductions in overall incidence of major amputations and related it to better control of diabetes (despite the increase in diabetes incidence) and better governmental public health strategies, such as creation of multidisciplinary teams for treatment of the diabetic foot and expansion of healthcare networks.^10^^,^ 14 ^,^ 15 ^,^ 29 ^-^ 31 The present cross-sectional and single-center study was unable to determine efficacy of public health initiatives with relation to amputations.

Limitations of the present retrospective study include a lack of additional clinical variables for study, such as obesity, HDL, LDL, and glycosylated hemoglobin levels, and medication usage, which is partially because data collection was dependent on correct completion of hospital medical records. Short and medium-term mortality rates could not be considered accurate, which removes a possible source of relevant data on the impact of each type of amputation on survival. Mortality data has almost never been available from the medical record, except in cases of intrahospital deaths and of patients who regularly returned to outpatients for follow-up, since many of the patients analyzed were later treated in their home towns, and notification services were not integrated. Rehabilitation (with prostheses) is another datum to which there was little access, because it is conducted at referral services in other towns. The prevalence rates of major comorbidities such as diabetes, hypertension, and kidney disease were higher in the present study than in other similar publications,^25^^,^ 32 ^-^ 34 which does not merely characterize the study population as at high risk, but also underscores the lack of public services for preventative healthcare in the geographic area studied.

In conclusion, it was observed that major and minor lower limb amputations exhibit different risk factors in the population group studied. Patients who underwent transtibial or transfemoral amputations were generally of advanced age, had more severe ischemia, anemia, sepsis, and/or stroke, whereas those who had amputations of toes or at the level of the foot had preserved perfusion and clear signs of diabetic neuropathy. Multicenter studies involving several surgical specialties related to amputations (vascular surgery, orthopedics, etc.) conducted with the objective of determination of short and medium-term mortality are fundamental to extending knowledge of the true impact of amputations on the patients under our care.
